# Embargo on Lion Hunting Trophies from West Africa: An Effective Measure or a Threat to Lion Conservation?

**DOI:** 10.1371/journal.pone.0155763

**Published:** 2016-05-16

**Authors:** Philippe Bouché, William Crosmary, Pierre Kafando, Benoit Doamba, Ferdinand Claude Kidjo, Cédric Vermeulen, Philippe Chardonnet

**Affiliations:** 1 University of Liège, Gembloux Agro-Bio-Tech, Unité de Gestion des Ressources Forestières et des Milieux naturels, Passage des Déportés, 2, B-5030 Gembloux, Belgium; 2 International Foundation for Wildlife Management, Rue Beaubourg 58, F-75003 Paris, France; 3 Direction de la Faune et des Chasse, Ministère de l’Environnement et du Développement Durable 03 BP 7044 Ouagadougou 03, Burkina Faso; 4 Direction Technique. Centre National pour la Gestion des Réserves de Faune (CENAGREF) 08 BP 0227 Cotonou, Bénin; University of Illinois at Urbana-Champaign, UNITED STATES

## Abstract

The W-Arly-Pendjari (WAP) ecosystem, shared among Benin, Burkina Faso and Niger, represents the last lion stronghold of West Africa. To assess the impact of trophy hunting on lion populations in hunting areas of the WAP, we analyzed trends in harvest rates from 1999 to 2014. We also investigated whether the hunting areas with higher initial hunting intensity experienced steeper declines in lion harvest between 1999 and 2014, and whether lion densities in hunting areas were lower than in national parks. Lion harvest rate remained overall constant in the WAP. At initial hunting intensities below 1.5 lions/1000km^2^, most hunting areas experienced an increase in lion harvest rate, although that increase was of lower magnitude for hunting areas with higher initial hunting intensity. The proportion of hunting areas that experienced a decline in lion harvest rate increased at initial hunting intensities above 1.5 lions/1000km^2^. In 2014, the lion population of the WAP was estimated with a spoor count at 418 (230–648) adults and sub-adult individuals, comparable to the 311 (123–498) individuals estimated in the previous 2012 spoor survey. We found no significant lion spoor density differences between national parks and hunting areas. Hunting areas with higher mean harvest rates did not have lower lion densities. The ratio of large adult males, females and sub-adults was similar between the national parks and the hunting areas. These results suggested that the lion population was not significantly affected by hunting in the WAP. We concluded that a quota of 1 lion/1000km^2^ would be sustainable for the WAP. Based on our results, an import embargo on lion trophies from the WAP would not be justified. It could ruin the incentive of local actors to conserve lions in hunting areas, and lead to a drastic reduction of lion range in West Africa.

## 1. Introduction

Lions in West Africa (*Panthera leo leo*) have been red listed as regionally endangered since 2004 [1]. They represent only 1.2% of the total estimated 35,000 wild African lions remaining [2,3]. Recent studies estimated that the West African lion population has lost 99% of its original habitat in the sub-region [3]. Lions of Asia and West, Central and North Africa seem to be genetically distinct from those of East and Southern Africa [4]. These findings increase the conservation importance of the West African lion population [3,5].

The main drivers of lion decline in West Africa have been the decline of wild prey due to unsustainable hunting [6] and/or the disappearance of perennial water points during the dry season [7,8], poaching for local medicine and other lion products, conflicts with local communities, increasing exposure to diseases from domestic animals [3,9,10,11], and habitat fragmentation and pollution from change in land use due to human demographic growth [12]. Change in land use also affects the local climate, decreasing surface water availability during the dry season due to lower and more irregular rainfalls since the 1960s [13].

Lion trophy hunting can also be a threat if not appropriately managed [14–18]. The dynamics of lion populations may be particularly sensitive to the removal of pride males because of social disruption and potential infanticide by incoming males [14–17]. Likewise, excessive trophy harvest may alter the sex ratio and ranging behavior, and eventually cause population decline [16,18–22].

During the last decade, there has been growing concern about the status of the African lion, with some parties advocating for a listing on CITES Appendix I [1]. Since January 2016, lions of Central, North and West Africa are officially listed as endangered under the US Endangered Species Act [23]. In the same period initiatives arose to convince the European Union to ban lion trophy imports [22]. Some countries such as Australia and France have recently banned the import of lion trophies [24,25]. Likewise, several airline companies decided to ban transport of some big game trophies [26]. Both the USA and the EU are requiring more data and monitoring from lion range countries, and are setting up stricter regulations for lion trophy imports than in the past. As lion trophy hunting is largely dependent on foreign trophy hunting clients, it is likely that stricter import regulations will affect the local trophy hunting community and the financial capacity of governments and operators to conserve hunting areas. However, numerous experts are convinced that quota and age-limit reform of trophy hunting is a better option than an Endangered listing [27]. Recently the EU, concerned that the trophy hunting quota was excessive in West Africa, asked Benin and Burkina Faso to investigate whether lion trophy hunting was the sustainable in the W Arly Pendjari (WAP) ecosystem, and if not to reduce their annual trophy quota [28,29].

Little has been published about lion trophy hunting in West Africa and its potential impact on lion populations, probably because it is nominal in scope compared to the rest of Africa [30–32]. Game-viewing tourism is embryonic in West African savannahs due to the lack of international professional operators, the low attractiveness of some wildlife species and landscapes, the lack of tourism infrastructure and marketing. Trophy-hunting in the region currently seems to be the best way to secure these unattractive ecosystems, and therefore to conserve wildlife and lions [32]. Trophy hunting represents more than 99% of the equivalent 2 million Euros in taxes generated by the wildlife industry in the WAP [32]. The trophy-hunting industry has made significant efforts to increase wildlife densities and carrying capacity to sustain international trophy-hunting demand [32]. It generates revenues shared with wildlife management authorities and local communities. Part of the revenue is used to self-support the wildlife management capacity of the WAP ecosystem [32,33].

In this paper we evaluate the impact of trophy hunting by assessing the trends in lion harvest over the past 16 years in the WAP complex that harbors West Africa’s most important remaining large carnivore populations [3], and the last remaining area of regular lion trophy-hunting in West Africa. We also present the results of the 2014 lion population survey. Under the hypothesis of a detrimental impact of trophy hunting on the lion population of the WAP, we expected 1) a decline in harvest rates over time, such decline being more marked in hunting areas with higher initial hunting intensities; 2) lower lion densities in hunting areas that experienced higher harvest rates; and 3) lower lion densities in hunting areas compared to neighboring national parks; and 4) a declining lion population. We then discuss the need to adjust hunting quotas, and to maintain a minimum lion quota in order to sustain the management of the WAP ecosystem and to ensure the future of the lion in West Africa.

## 2. Methodology

### 2.1 Study area

**Fig 1:**
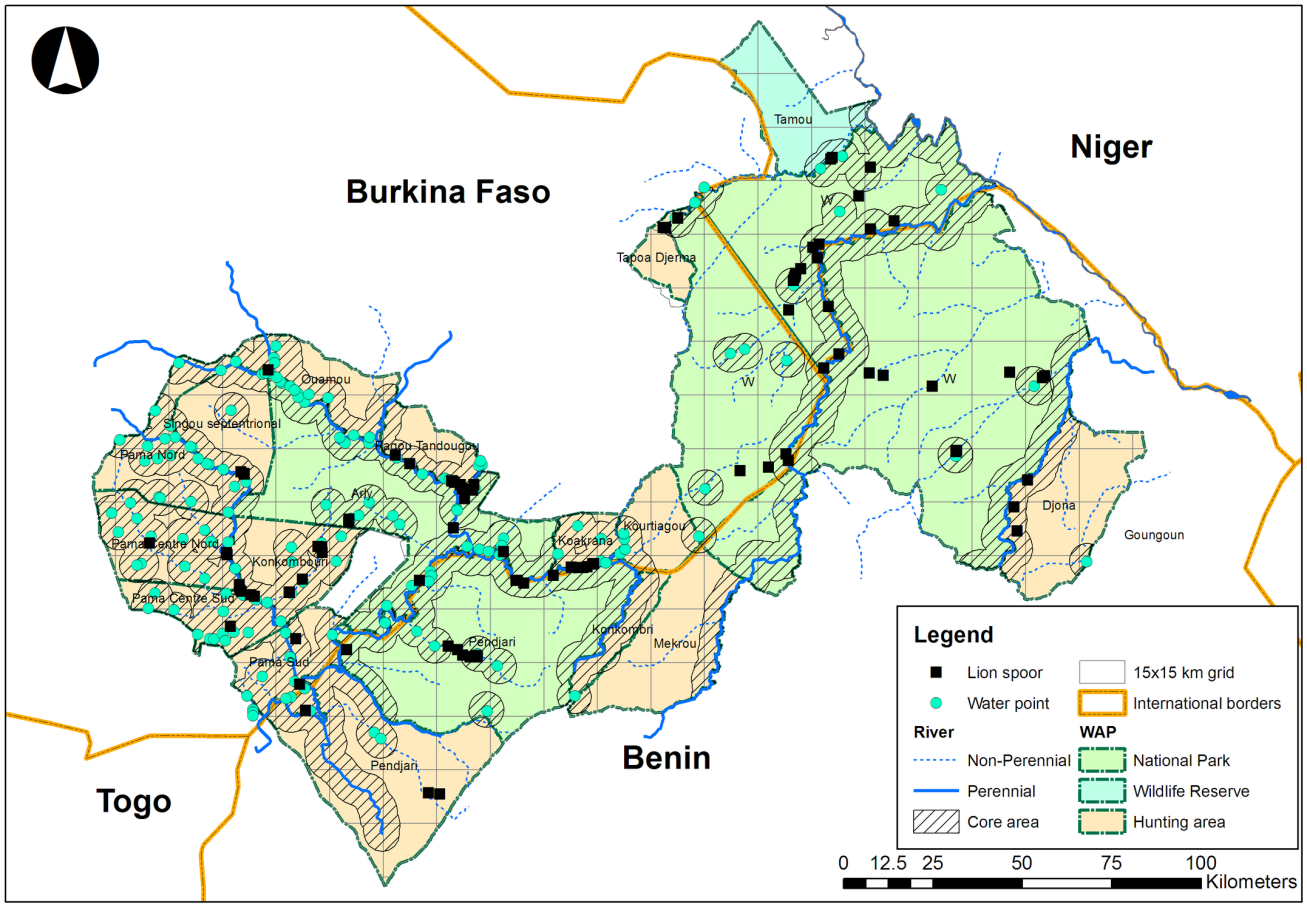
Distribution of fresh lion spoor in the WAP lion’s range. The core area to wildlife in the dry season represents a 5 km buffer around all remaining water points and known springs during the dry season.

The WAP covers an area of 33,000 km^2^ located in West Africa between 9°95 and 12°85 North latitude and between 0°40 and 3°40 West longitude, shared by Benin, Burkina Faso and Niger. The range of the lion in the WAP is 26,038 km^2^. Up to the end of 2013 the national parks and hunting areas covered respectively 55.7% and 43.5% of the WAP. The current ecosystem and lion range is a patchwork of national parks (58.6%), hunting areas (40.6%) and game reserves (0.8%) (Fig 1) following the 2014 extension of the Arly National Park to some former hunting areas, which doubled the size of the Park and reshaped two neighboring hunting areas (Konkombouri and Singou) (S1 and S2 Tables). The WAP currently harbors 88% of West African lions [3].

The weather is characterized by three seasons: a dry, cool season from November to late February; a wet, hot season from March to May; and a rainy season between June and October. The Harmattan, a dry, cold wind from the North East, blows during the dry cool season, drying up the pastures. During the rainy season a monsoon wind blows from the southwest. The rainfall follows a unimodal pattern and varies between 685 mm (north) and 1,100 mm (south). The average annual temperature is 28°C [34]. The landscape is dominated by *Combretum* and *Terminalia* savannah woodlands, with forest galleries along the main rivers bordered in some places by *Andropogon* and *Hyparrhenia* grassy floodplains [34].

### 2.2 Benin and Burkina Faso lion hunting regulations

Hunting areas leased to private operators currently represent 40.6% of the WAP Ecosystem (Fig 1). These areas are leased to persons or companies in the country. Lion trophy hunting occurs exclusively in Benin and Burkina Faso, in 16 hunting areas of 250 to 1800 km^2^ allocated by the governments to trophy-hunting operators since 2001 and 1996 respectively. The concessions boundaries were fixed since with the exception of the 2 hunting blocks (Konkombouri and Singou) affected by the Arly National Park extension in 2014. The leasing length was of ten years in Burkina Faso between 1996 and 2006, and of 20 years since 2006 [35]. In Benin the leasing length was of 5 years renewable up to 2013, and shifted to 10 years renewable in 2014 [36]. This concession system was set up after the failure of former wildlife management and hunting policies [37]. Before 1996 in Burkina Faso and 2001 in Benin, hunting areas did not exist and the land dedicated to hunting was poorly managed (few roads, no water points). The states had very few resources to control the hunts in such large areas and wildlife density declined drastically between the 1980s and the early 1990s [38]. This convinced the states in 1996 to concede hunting blocks to private operators with a dedicated and responsible manager for each block that pays rental fees but that is also responsible for managing the area. This resulted in wildlife numbers strongly increased these last decades [39]. The states have devolved management responsibility for the hunting areas to the operators, except for some law enforcement activities and quota setting. Hunting areas are allocated through a public bidding process where the candidate is selected according to the best management and investment plan. Private operators’ investments in terms of water points, roads, camps etc. are critical for the status of wildlife populations [36].

Among their duties, the hunting operators are obligated to provide ¾ of the meat harvested from hunting to neighboring communities. In Burkina Faso, the concessionaires pay the half of the concession rental fee (1.14 Euro/km^2^) to a community fund named FIC (*Fonds d’Intérêt Communautaire*) [36]. In Benin the 30% of hunting revenues are paid to the AVIGREF (*Association Villageoise de Gestion des Ressources Fauniques*) [35]. Moreover all camps employ personnel from the local communities for housekeeping the camps and for wildlife tracking during the hunts. Local communities manage also small hunting area of few km^2^ dedicated to bird hunting. Bird hunting activities depend strongly on the large game hunting clients, who often spend a day hunting birds at the end of their large game-hunting safari. They pay a daily fee and the service of local trackers [36].

**Fig 2:**
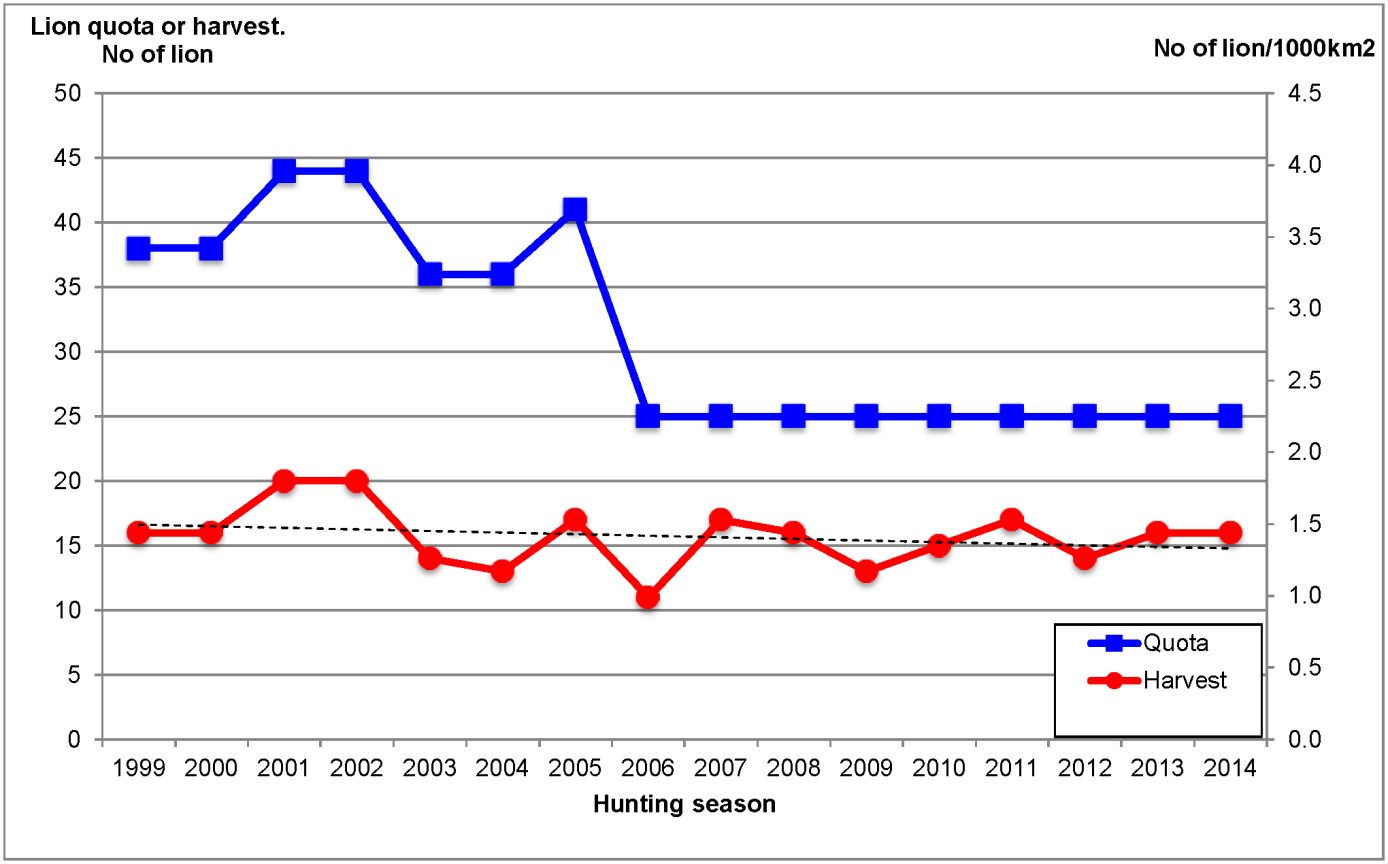
Trends of the total annual lion quota or harvest rate (number of lions /1000 km^2^) between the first year of lion hunting and 2014 in the WAP (Benin and Burkina Faso) (see text). A hunting season ran from December to May. Every season corresponds to a year. By convention the season of a particular year covers the month of December of the previous year. For example the season 1999 covers the months from December 1998 to May 1999. In Burkina Faso, data were available since 1999, just 2 years after the implementation of the concession system launched in 1996–97 season. Therefore we calculated the initial hunting intensity for the 1999–2001 period with one exception: Singou, which received its first quota in 2000 (S1 table). Benin started the concession system in 2001–02 (data per area are available since this year) but established a moratorium on lion hunting in 2003 and 2004. Therefore the initial hunting intensity was calculated for 2005–2007 except for Djona that started the harvest in 2010 (S1 Table).

The global lion quota in the WAP has increased from 38 in the late 1990s to 44 lions in 2001. Since 2006 the annual quota has been set at 25 (Fig 2). It must be noted that Benin imposed a lion quota of zero in 2003 and 2004. The quotas are set each year with a participative (all stakeholders) and exhaustive (including all available information sources) method [40,41]. The regulations limit off-take to male lions and restrict the hunting method to tracking. Baiting is not permitted. Since 2005, no lion-hunting quota has been allocated to national or resident expatriate hunters. The lion hunt activity is therefore dependent upon international clients, mainly from the West (Western Europe and the US), but also increasingly from Russia [42].

Benin and Burkina Faso ministries in charge of wildlife and protected areas have taken several measures to ensure that local hunting practices conform to international practices [43]. Since 2013 Benin and Burkina Faso have developed an age-based restriction to male lions ≥ 6 years old that are not part of a known pride [43–44]. All trophies must be presented to the wildlife authorities for a trophy quality check by an independent conservation NGO or a university prior to delivery of any export permit in compliance with the CITES rules. Operators, local officers and skinners receive training about collecting information from trophies according to international standards [45]. Underage trophies are to be forfeited and a penalty assessed to the hunting guide that allowed the shooting of non-compliant trophies.

In addition, the West African association of trophy hunting operators raised the revenue value of the lion hunt by changing the minimum 15-day hunt to 21 days and by increasing the minimum hunting package to 15,000 Euros, starting with the 2015–16 hunting season. The duration of trophy-hunting journeys will be verified by the states. Benin and Burkina Faso, which had the lowest lion hunt permit fees in the trophy-hunting market, agreed to increase the trophy fee to 4,600 Euros, commencing with the 2015–16 hunting season [43].

As the WAP lion population is shared by Benin, Burkina Faso and Niger, the three countries adopted a joint action plan for the conservation of large carnivores in the WAP according to IUCN guidelines [43–46] in addition to the national conservation plan in Benin [47]. The regional action plan outlines common management measures for conserving large carnivores in the entire ecosystem across all borders. This plan includes contributions from the three countries, NGOs and trophy operators.

### 2.3 Lion harvest

Because overhunting could be a cause of a decline in harvest rate over time, we compiled lion trophy harvest data for the past 16 years (1999–2014) from the Benin and Burkina Faso wildlife authorities [48–49]. To investigate the trends in lion harvest at the scale of the WAP, we ran a simple linear regression of the lion harvest rate, i.e. number of lions harvested/1000 km^2^ in the hunting areas, against the years, from 1999 to 2014.

At the scale of the hunting areas, we tested for a decline in harvest rates over time, expected to be more marked in hunting areas where harvest rates were the highest at the beginning of the study period. Therefore, we first defined the initial hunting intensity as the average annual number of lions harvested per 1000 km^2^ during the first three years of exploitation. We then regressed the harvest rates against the years, i.e., between 1999 and 2014, and defined the annual percentage change in lion harvest as the regression coefficient divided by the initial hunting intensity [18]. The annual percentage change in lion harvest is a measure of the annual change in lion harvest rate in proportion to the initial hunting intensity. It enabled us to compare the magnitude of the annual changes in lion harvest rates among hunting areas of different initial hunting intensities. Because the rate of change approaches zero at high initial hunting intensities, we log-transformed all data sets where initial hunting intensities exceeded 3 trophies per 1000 km^2^ per year [18]. Various regression models were then performed (linear, quadratic, negative exponential, non-linear model and piecewise polynomial). The best model was selected using the concordance criterion [50].

To test whether lion densities were lower in hunting areas with higher harvest rates, we performed a linear regression between lion densities and harvest rates.

Recommended future quotas were defined according to the trends in lion harvest rates [18], and according to the harvest rates recommended in the literature [16,51].

### 2.4 Lion survey

We carried out a lion spoor count [52] in the WAP. The number of spoors per km provides an index termed “track density”. For every large carnivore species, the track density is highly correlated with actual population density [52]. In order to spread transects homogeneously, a 15x15 km grid was superimposed over the WAP boundaries. In each grid cell, a 15 km transect was selected along a road where a road network existed.

Five teams were trained to collect data prior to the survey. Each team had to cover transects in a given park and/or contiguous hunting areas. The team consistently drove the transects in a car between 7:00 and 9:00 am at the speed of 10 km/h. One hundred transects totaling 1,492.6 km were completed between 22 February and 7 April 2014.

Each time a spoor was encountered, the location was recorded with a GPS, and walking direction and quality of the substrate were recorded according to [53]. The species was identified, the spoor photographed, and spoor freshness evaluated (Fresh spoor < 24 hours, Recent 24–72 hours, Old >72hours). Only fresh spoors were analyzed for estimating the lion population.

Transects could be partially or totally abandoned when it had rained during the night or in the morning or when another vehicle had passed before the counting team’s vehicle, possibly erasing the spoors.

Spoor identification and freshness were rechecked from photographs by two independent observers. Only fresh spoors from adults and sub-adults were used for the analysis. Spoors from young lions (<1 year old) were discarded because their mortality is high (50–90%) [54]. The track density was converted to population density [52].

Because hunting can reduce lion populations, we tested whether lion spoor densities were higher in national parks than in the hunting areas. As lions are water dependent [55,56], we also tested for differences in lion densities in areas close to water (<5km) versus areas far from water (>5km). We used the Mann Whitney test to look for statistical differences [57]. WAP water distribution and availability data were compiled from [58,59] and visually confirmed against 2013 and 2014 Google Earth images, for all the remaining water points along main watercourses and known springs away from rivers in the period of the survey.

We compared the 2014 lion estimate with a previous lion estimate from 2012 [53] using a d test [57].

Spoor photographs were utilized to determine large adult males’ spoors (spoor length ≥12 cm) to calculate the ratio of large males in comparison to females and sub-adults.

## 3. Results

### 3.1 Lion harvest

Across the hunting areas of the WAP, the number of lions harvested each season ranged from 1 to 2 lions /1000 km^2^ (mean: 1.4 ± 0.2 lion/1000 km^2^). The lion harvests were below the quota at each season (mean: 2.8 ± 0.7 lions/1000 km^2^) (Fig 2). Harvest rate did not significantly change over the years (y = -0.0143x+1.524; R^2^ = 0.07; P>0.05) (Fig 2).

**Fig 3:**
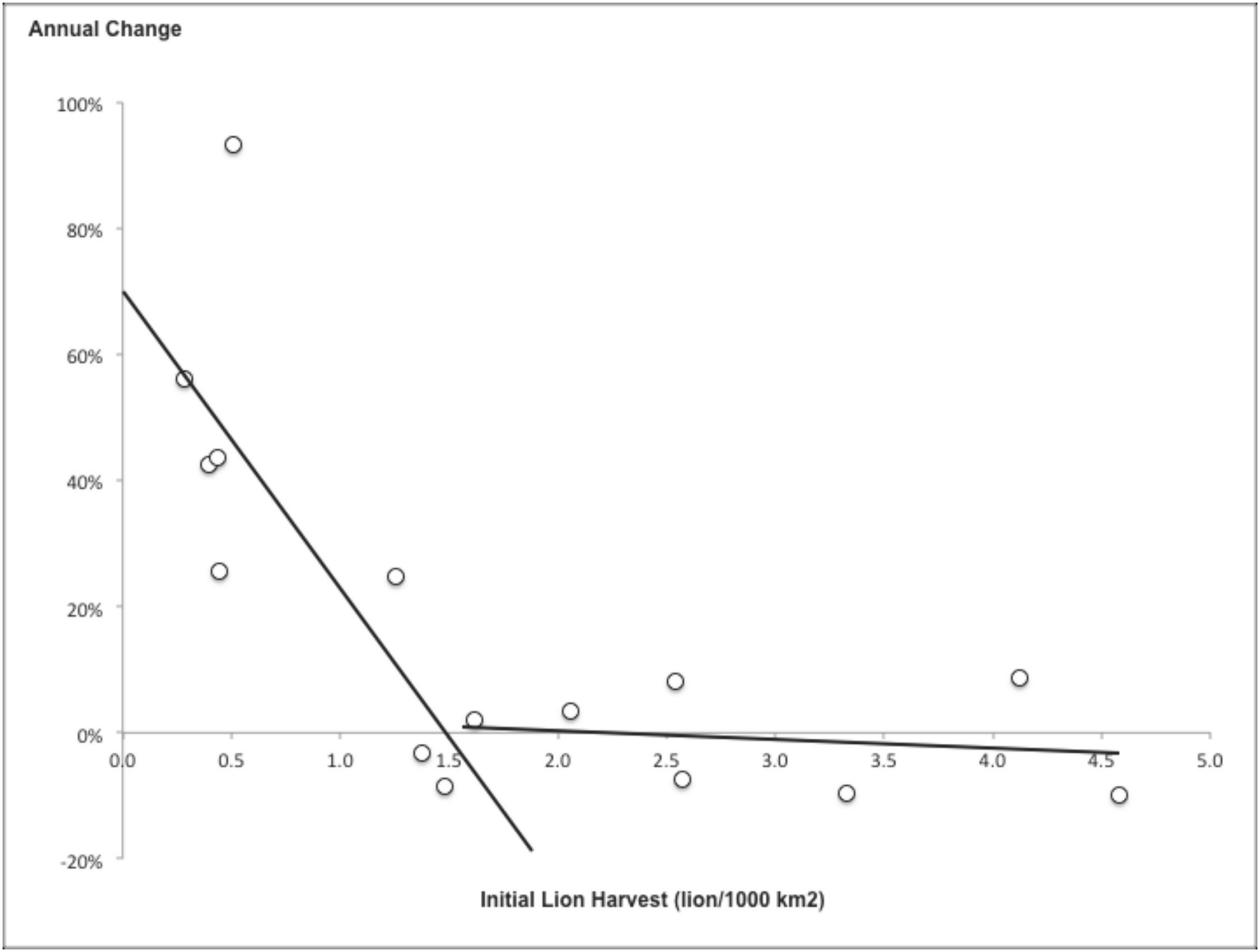
Annual percentage change in the lion harvest between 1999–2014, vs. initial hunting intensity. Each point represents a hunting area. A single area (Mékrou) had an annual percentage change of -45% with an initial hunting intensity of 0.6 lion/year. It was considered as an outlier and discarded from the analysis.

As expected, lion harvest rates declined with increasing initial hunting intensities (Fig 3). However contrary to what we expected, most hunting areas, ten out of sixteen, experienced an increase in lion harvest during the study period, with the highest magnitudes of increase at initial hunting intensities below ca. 1.5 lions / 1000 km^2^ (Table 1, Fig 3). The hunting areas that experienced a decline in lion harvest were those for which initial hunting intensities were above ca. 1.5 lions / 1000 km^2^ (Fig 3).

**Table 1:** Proportional change in harvest of lions vs. initial average harvest regression models, value of the concordance criterion (CC), Equation, value of R^2^ and P. The selected model is indicated in bold.

Type of regression	CC	Model	R^2^	P
Linear	0.63	y = 0.4540–0.1490 x	0.48	0.003
Quadratic	0.79	y = 0.6859–0.4945 x+0.07688 x^2^	0.67	0.016
Negative exponential	0.77	y = 0.969484. exp^(-1.56371x)^	0.62	0.006
Non linear	0.72	y = 0.179383 x^(-1.06983)^	-	-
**Piecewise**	0.83	y = 0.6999–0.4569 x and y = 0.03959–0.01702 x	0.70	0.005

### 3.2 Lion survey

In total, 97 fresh spoors of adult and sub-adult lions were recorded. The mean substrate quality recorded was 2.38, suitable for detecting spoor along the transects (Table 2).

**Table 2:** Results of the lion count. Area in km^2^, Number of transects, Transects length in km, Substrate quality, Number of fresh spoors observed, Track density (N/100 km), estimated lion population density (N/100 km^2^), lion estimate (N), Coefficient of variation (CV) in %, minimum and maximum 95% Confidence Interval (CI) in each stratum of < and > 5 km from water and for the entire W Arly Pendjari Ecosystem. Details per area is given in S2 Table.

Stratum	Area (km^2^)	Number of transects	Transects' length (km)	Substrate quality	Number of spoors	Track density	Lion density	N	CV%	95% CI	
						N/100 km	D (N/100 km^2^)			min	max
< 5 km from water	11843	70	1062.6	2.34	87	8.19	2.58	305	13%	228	383
> 5 km from water	14195	30	430.0	2.72	10	2.33	0.80	113	27%	53	173
W Arly Pendjari	26038	100	1492.6	2.38	97	6.50	1.61	418	23%	230	607

**Fig 4:**
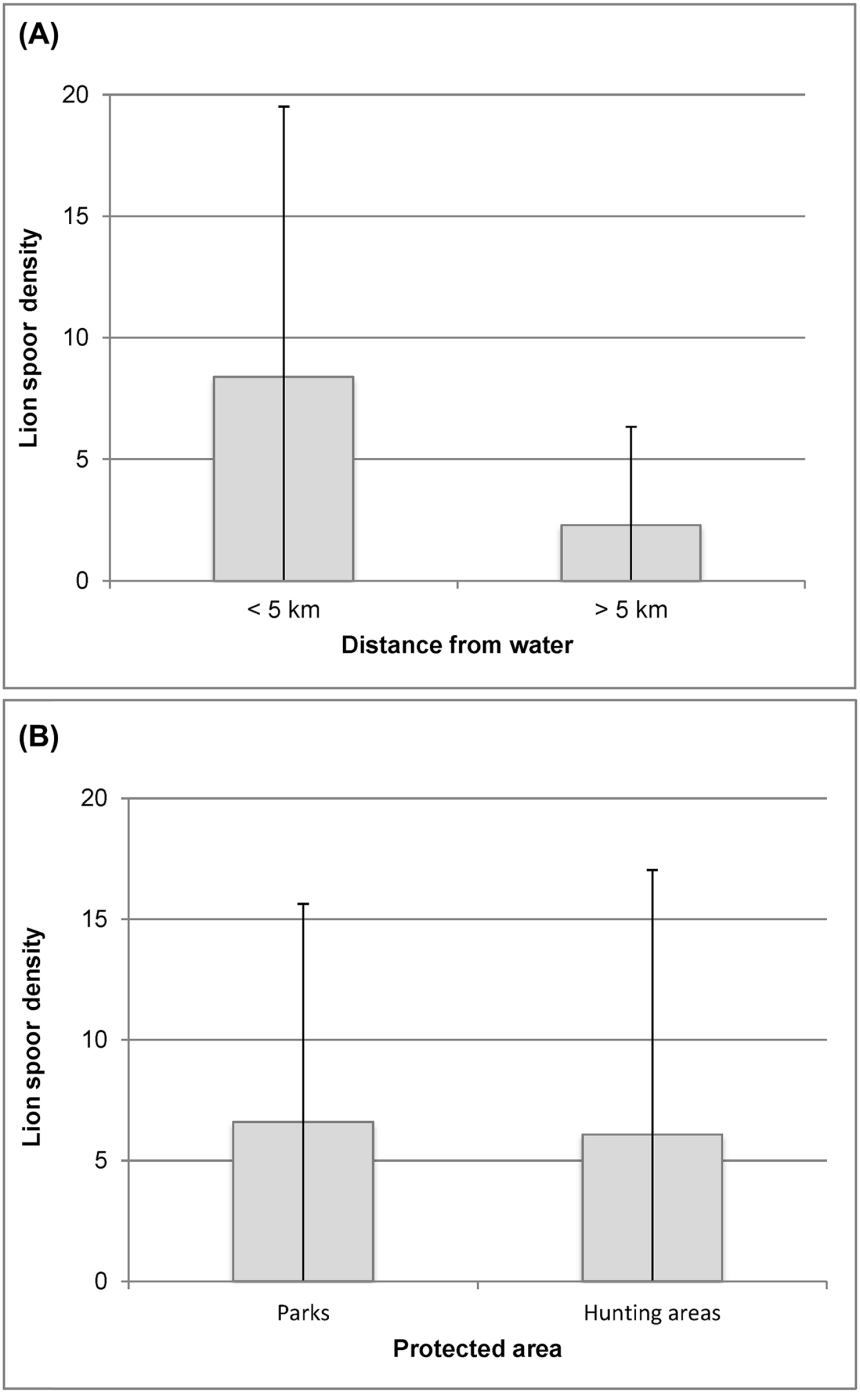
Lion spoor densities (N/100km): (a) at < and > 5 km from water in the WAP (the vertical lines represent the standard error); (b) in national parks and hunting areas of the WAP (the vertical lines represent the standard error).

Spoor densities significantly differed between strata, i.e. < versus >5 km from water (*U* = 1390; *z* = 2.80; n_1_ = 70; n_2_ = 30; *P* < 0.01). Ninety percent (90%) of all fresh spoors recorded fell < 5 km from water (Fig 4a). However, there was no significant difference in spoor densities between parks and hunting areas of the WAP (*U* = 1430; *z* = 0.83; n_1_ = 56; n_2_ = 47; *P* > 0.05) (Fig 4b).

The lion population was estimated at 418 (95% CI: 230–648) adult and sub-adult individuals (Table 2). In the WAP, 217 lions (52% of the population; 1.7 ± 0.35 lions/100km^2^) were estimated in Burkina Faso while 152 lions (1.4 ± 0.39 lions/100km^2^) were estimated in Benin and 49 in Niger (1.95 ± 1.08 lions/100km^2^).

In the WAP, the ratio of large males to females and sub-adults in parks (16 large males vs. 35 females and sub-adults, ratio = 0.46) did not significantly differ from that in hunting areas (13 large males vs. 33 females and sub-adults ratio = 0.39) (χ2 = 0.11; df. = 1; P > 0.05). The WAP population of large males was estimated at 168 individuals.

**Fig 5:**
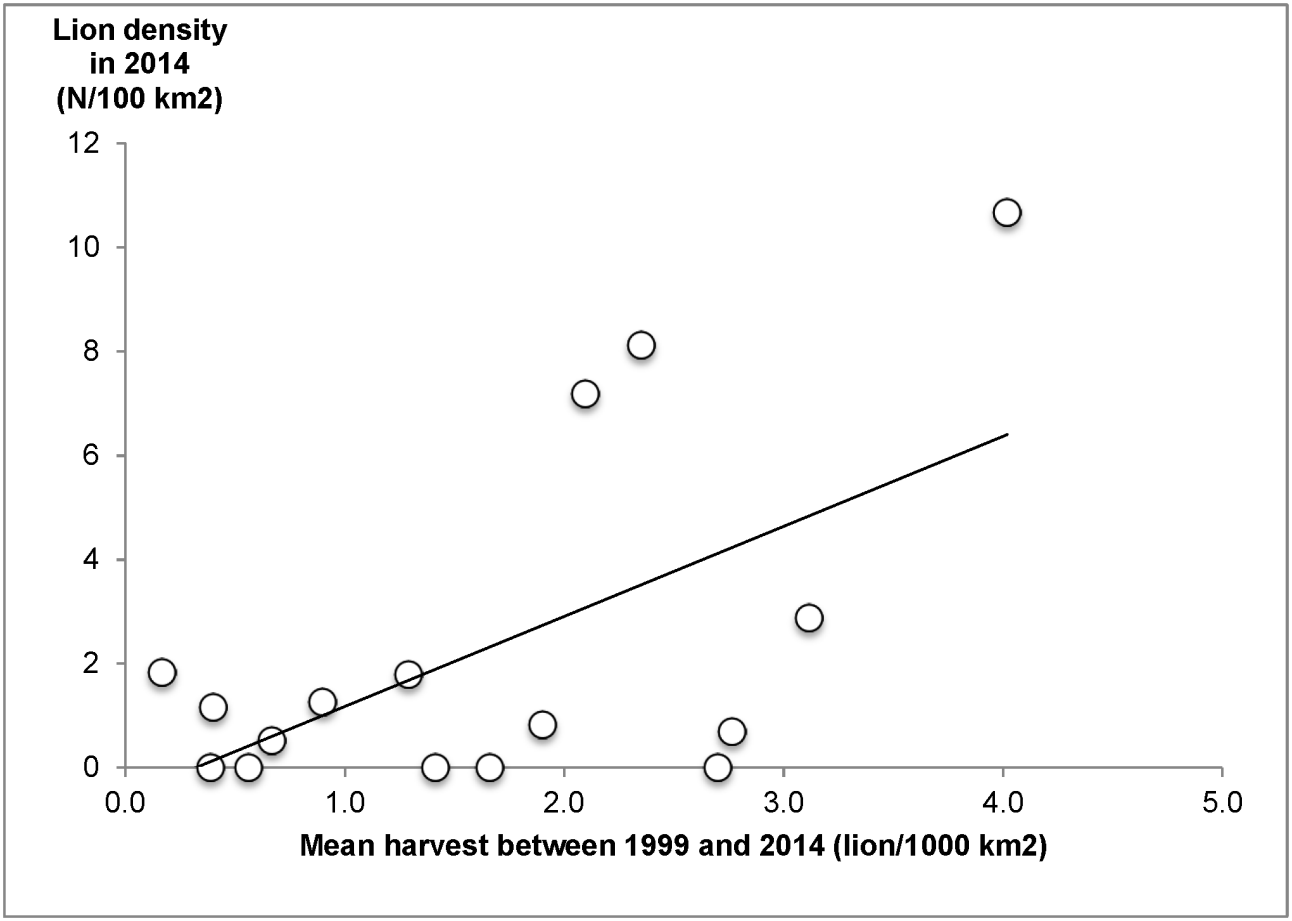
Lion density in 2014 (N/100 km^2^), vs. the mean of 16 years harvest between 1999 and 2014 (mean number of lions harvested/1000 km^2^). Each point represents a hunting area.

Contrary to what we expected, the hunting areas that experienced higher harvest rates had the highest lion densities (y = 1.7314x−0.5591; R^2^ = 0.35; *P ≤ 0.05; Fig 5).

## 4. Discussion

### 4.1. Lion survey and harvest

The lion harvest in the WAP has been stable for the past 16 years (Fig 2). This finding differs from those of other studies in Tanzania and northern Cameroon for instance, where lion harvests decreased in almost every ecosystem and where the number of trophies harvested was irregular from one year to another [18,20]. This could be explained by the higher initial hunting intensities (between 2 and 4 harvested lions/1000 km^2^) recorded in some Tanzanian hunting areas. Interestingly, the hunting areas in Tanzania with lower initial hunting intensities, i.e. between 0.5 and 2 lions/ 1000km^2^, showed stable lion harvests [18], similar to the harvest rate we found in the WAP (1.4 ± 0.2 lions/ 1000 km^2^) (Fig 2).

Most hunting areas at initial hunting intensities below 1.5 lions/1000km^2^ harvested more lions in 2014 than in 1999. However as expected, the hunting areas with higher initial hunting intensities, i.e. above 1.5 lions/1000km^2^, failed to increase or to maintain their lion harvest rate between 1999 and 2014 (Fig 3).

From the 2014 lion spoor counts, we observed no significant difference in densities of lion between the national parks and the neighboring hunting areas of the WAP complex. Lion numbers in the WAP increased from 311 to 418, ca. 30%, between 2012 (the only previous comparable lion survey [53]) and 2014. However, we found no statistical difference (*d* test = 0.63; P>0.05) between these two years, probably because of the large confident intervals associated to these lion number estimates, 311 [123–498] in 2012 and 418 [230–607] in 2014.

Interestingly, lion densities were higher in hunting areas experiencing higher average harvest rates. These hunting areas generally benefit from favorable management, and/or abundant water availability, usually hosting more large herbivores, and as a consequence possibly more lions [39,58–59]. Therefore, any negative impacts on the lion population arising from hunting appear to be either minimal or to be offset by management that protects and fosters populations of both lions and their prey. Moreover, the ratio of large males versus females and sub-adults was similar in parks and hunting areas. Again this suggests that in the WAP, the impact of trophy hunting on lion population demography has been relatively moderate compared to other African hunting areas [16,19–21]. Nonetheless, it has been reported that the hunting zones in the Benin part of the WAP had smaller lion groups, more observations of single lions and a sex ratio more skewed towards males [8]. Overall, these results suggest that trophy hunting in the WAP did not affect densities of lion over the last 16 years. No lions live in the neighboring non-protected areas of the WAP. Therefore hunting areas seem to be as effective as national parks in protecting lions.

In Tanzania, when initial hunting intensities were above ca. 1–1.5 lion / 1000km^2^ in Selous Game Reserve, and ca. 0.5 lion / 1000km^2^ in the other ecosystems, most hunting areas experienced declines in harvest rates [18]. This led the authors to recommend a quota of no more than 1 lion/1000km^2^ in Selous Game Reserve, and 0.5 lion/1000km^2^ in the other ecosystems. In the WAP Complex, most hunting areas experienced an increase in harvest rates during the study period, even at initial hunting intensities higher than 1.5 lion/1000km^2^. However, for the hunting areas of the WAP, (10,341 km^2^), as a precaution we would recommend an annual quota corresponding to a harvest rate that does not exceed 1 lion/1000km^2^. Therefore the WAP quota would be a maximum of 10 male lions per year (6 in Burkina Faso and 4 in Benin). This represents 6% of the large male numbers of the WAP (168 individuals), or 2.4% of the WAP lion population (418 adults and sub-adults), which is in line with the recommendations to not harvest more than 10% of a population’s adult males [16], or not more than 3% of the population size [51]. At the scale of the WAP (parks and hunting zones), the global lion harvest rate would represent 0.4 lion /1000 km^2^. This harvest rate is less than 0.5 lion/1000 km^2^, which is the most conservative published hunting quota rate [18].

### 4.2 Conservation issues

Inside ecosystems where hunting blocks exist, a decline in lion numbers has been at least partly attributed to legal lion hunting [16,18]. It is however remarkable to notice that in West Africa, on the contrary, lions disappeared first from regions where hunting areas and the private sector are absent or inactive (e.g. Boucle du Baoulé in Mali, Comoé in Côte d’Ivoire, Mole in Ghana, Niokolo Koba in Senegal, Yankari in Nigeria) [3]. The WAP is currently the last large stronghold in West Africa where a viable lion population (418 individuals) remains. The national wildlife authorities that manage many West African national parks have failed in many cases to protect them, often due to the lack of available resources from local governments, to low or non-existent tourism activity and to lack of support from international conservation NGOs [3,60,61]. Many areas are not truly protected and act only as ‘paper parks’ [11].

Our results show that in the WAP the lion density was higher close to water points. This suggests that the maintenance of perennial water points is a fundamental step for conserving lions and also their prey. Other studies suggest [58,59] that in some parks like W parks (Fig 1), the first driver of wild population depletion is not poaching, as many think, but the unequal distribution of water that limits the suitable habitat to a few km from the last remaining rivers during the dry season. Permanent water points therefore need to be managed to sustain higher wildlife densities. Since 1996 private hunting operators in Burkina Faso have created and maintained about 30 new water points in addition to natural perennial water points and rivers. This corresponds to an increase of 78% of the core area available to wildlife during the dry season since the beginning of the concession rental system in 1996. Today, the core area available to wildlife represents 78% of the size of the hunting areas on the Burkina side (Fig 1) [36,58,59].

The lion is the most valuable species marketed in trophy-hunting areas in the WAP, as in other countries [16,22]. Lion trophy hunting generates the highest revenue of all trophy species [22]. Unwarranted restrictions on lion hunting may reduce tolerance for lion in communities where local people benefit from trophy hunting [22] and reduce funds available for anti-poaching and management activities.

We have here demonstrated that the impact of lion hunting for the past 16 years in the WAP has not been detrimental to the lion population. Considering the trends in lion harvest rates during this period, adjusting the quota to 1 lion/1000km^2^ would be sustainable. If lion trophy hunting is unduly reduced by import restrictions, the loss of revenue will affect the WAP’s self-supporting financial capacities and reduce the competitiveness of wildlife-based land uses relative to ecologically unfavorable alternatives [22]. The depreciation of the lion’s value and the loss of incentive for trophy-hunting operators to protect them will no longer prevent cattle herders from eliminating their enemies. Even worse, some trophy-hunting operators may view the lion more as a competitor for the herbivore trophy species sought by their hunter customers. Therefore, the hunting areas of the WAP (10,584 km^2^, 40.6% of the current lion range) will become less favorable to lions. In the meantime experts plead for the urgent need for very large and well-protected areas to assure the survival of lions and other threatened large mammals [3]. Maintaining the full WAP area suitable for lions is critical to conserve a viable population (250 to 500 individuals) in the long term [2,9,62]. An import ban would drastically reduce the WAP lion’s range and could have the opposite consequence than the one desired. Import bans could precipitate the extinction of the West African lion.

## Supporting Information

S1 Table Lion Quota (Q) and Harvest (H) in hunting zones of the WAP. (XLSX)

S2 Table 2014 Lion survey results in the hunting areas of the WAP. (XLSX)
